# Veterinary Titles

**Published:** 1893-12

**Authors:** 


### EDITORIAL.

### VETERINARY TITLES.

"Thou art not so long by the head as honorificabilitudinitatibus." (Shakespeare.) —*Love's Labour's Lost, V. 1.*

When Hercules found the hind, with his cart stuck in the mire, bellowing for help, he told him that the "Lord helps those who help themselves," or something of that sort. The God who was first appealed to by the veterinary profession for help, in the preliminary steps of "elevating" their guild, must have given the same advice, which the Horse Doctor and Cow Leech followed with a vengeance. An inspection of the "Veterinary Register" at a County Clerk's office does not seem to indicate that the inscriber's knowledge of the use of the letters of the alphabet and meaning of their combinations has any influence upon the number of those which he suffixes to his name, with or without reason or right. To make things easy, however, the legislators who formulate the restrictions upon freedom which civilized communities call "laws," and their henchmen, the executors of these laws, who find a dollar or so fee with each registration, do not bother much about the "Vet." The United States Government, New York, Pennsylvania, and other great Commonwealths, receive and recognize as peers, accept testimony in courts from, and appoint to offices requiring professional skill, man for man alike, whether his credentials are under the seal of an honorable institution of learning, or his signature is John X Doe. (HIS / MARK.)

Great Britain has a certain uniformity in title, and the graduate from London, Edinburgh and Glasgow alike, is an M.R C.V.S. (Member of the Royal College of Veterinary Surgeons), recognized everywhere as a legally qualified practitioner. The Royal College publishes a register in which can be found a record of the graduates, IF, in their peregrinations to Canada, the United States and elsewhere, they remember from time to time to remit a five dollar gold piece to their Alma Mater or whoever composes the Board of Registry, but who is dropped from the rolls if he fails to pay.

America, being a larger country, gives the "Vet" a more liberal and larger choice of titles. In New York, the two schools, each of which has "the only legal charter in the State to grant diplomas," make Veterinary Surgeons, V.Ss., and Doctors of

Veterinary Surgery, D.V.Ss., respectively. Harvard and the University of Pennsylvania—both great centres of learning, appreciated that medicine is a more comprehensive word than, and *includes*, surgery, so they make Doctors of Veterinary Medicine, but the classical teachers of one of the universities were at fault in their Latin and the one makes V.M.Ds., while the other has M.D.Vs., and the Iowa State Agricultural College compromises by turning out D.V.Ms. Montreal, when it found shelter under the Ægis of McGill University as a Faculty of Comparative Medicine and Veterinary Science, became osteo-porotic and elevated the V.S. to D.V.S., but not to a plain D. V. *Surgery*, like a New Yorker, but to a D. V. *Science*. Chicago, in view of the white palaces and thronging multitudes which Columbus brought to the lake shore, became positively megalo-cephalic, and turned out M.D.Cs. (*Doctors of Comparative Medicine*) instead of simple V.Ss. as formerly. Graduates from French schools translate their diploma of "*Vétérinaire*" into *Veterinarian*, while the German "*Thierarzt*," or Animal Doctor, can make his own choice from American abundance in nomenclature.

The catalogues of some of the Veterinary schools do not give the degree conferred, and copies of their diplomas are not available, so that corrections to the following list is requested :

| INSTITUTION. | DEGREE. | ABBREV. |
|---|---|---|
| University of Pennsylvania. | Veterinariæ Medicinæ Doctor. - | V. M. D. |
| Harvard University. - | (?) - - - | M. D. V. |
| Iowa Agricultural College. - | (?) - - - | D. V. M. |
| McGill University (Montreal). | Doctor of Veterinary Science. - | D. V. S. |
| American Veterinary College. | Doctor of Veterinary Surgery. - - | D. V. S. |
| Ohio Veterinary College. | Doctor of Veterinary Science. - | D. V. S. |
| Detroit Veterinary College. | Doctor of Veterinary Science. - - | D. V. S. |
| National Veterinary College. | Doctor of Veterinary Science. - | D. V. S. |
| Chicago Veterinary College. | Doctor of Comparative Medicine. - | M. D. C. |
| New York College of Veterinary Surgeons. | Veterinary Surgeon. - - | V. S. |
| Ontario Veterinary College. | Veterinary Surgeon. - - | V. S. |
| Kansas City Veterinary College. | (?) | |
| Cornell University. | Bachelor of Veterinary Science. - | B. V. S. |
| Indianapolis Veterinary College. | Veterinary Surgeon. - - - | V. S. |
| Royal College, London.<br>Dicks', Edinburgh.<br>New, Edinburgh.<br>Glasgow. | - - - - | M. R. C. V. S. |

When titles become further complicated by the possessors having an M.D., a Ph.D., Sc.D., or an honorary title from the Royal College, the abracadabra of the evidences of learning becomes formidable.

Perhaps as long as the course of instruction in the various schools ranges from a few months to three years, it is well to have the titles distinctive, so that the initiate may understand that :

S. V. D.=Instruction for one winter+$87 fee.

D. S. V.=24 months' hard study+rigid examinations.

S. V. D.+D. S. V.=No recognition by Government.

H. H. H.=Spavin cure protected by Government Patent.

John Smith (painter by trade)=Government Cattle Inspector

It may not be time to ask for a unity of title until the regularly and properly educated veterinarian is legally recognized by the Government. We think, however, that this question is of sufficient importance to be discussed by the United States Veterinary Medical Association. The latter is a body which must become the controlling institution of the profession at large. It is a voluntary association, organized for the general good and now entirely free from any local prejudice or affiliation with schools or colleges, which latter are always influenced more or less in any changes by the pecuniary result. The United States Association has recently granted to its members the privilege of using its seal on stationery, which was a wise move so long as it guarantees the professional and reputable standing of its members ; it would be fortunate if it could adopt some short, comprehensive, distinctive title.

